# Brassinin from *Brassica campestris* L. inhibits colorectal cancer by inducing p62/NRF2/GPX4‐regulated ferroptosis

**DOI:** 10.1002/ame2.12521

**Published:** 2025-01-23

**Authors:** Shi‐Yuan Wen, Rui‐Rui Gao, Yan‐Yan Chen, Yi‐Jie Wang, Xin‐Tong Wang, Hai‐Xin Liu

**Affiliations:** ^1^ College of Basic Medical Sciences, Shanxi Medical University Taiyuan China; ^2^ College of Traditional Chinese Medicine and Food Engineering, Shanxi University of Chinese Medicine Taiyuan China; ^3^ School of Medicine Jiangsu University Zhenjiang China

**Keywords:** Brassinin, colorectal cancer, ferroptosis, GPX4, NRF2

## Abstract

**Background:**

Indole phytoalexins, plant‐derived compounds present in cruciferous vegetables, have demonstrated anticancer properties. Brassinin (BSN), derived from *Brassica campestris* L. var. campestris, is known for its potent antitumor effects on various cancers. However, the role of ferroptosis in regulating the antitumor effects of BSN has not been fully elucidated.

**Methods:**

The components of *B. campestris* L. against colorectal cancer (CRC) were analyzed by network pharmacology. CCK‐8 assay and colony formation assay detected cell viability induced by BSN. Molecular docking verified the binding of BSN to the target protein. Western blot and reverse transcription–quantitative polymerase chain reaction (RT‐qPCR) assay revealed whether BSN can inactivate the NRF2 signaling and inhibit the expression of p62 and HO‐1. The RKO‐xenograft tumor models were established and then were treated by 75 or 150 mg/kg BSN to verify the antitumor efficacy and side effects of BSN.

**Results:**

Network pharmacology suggested that BSN is the most important component of *B. campestris* L. against CRC. BSN inhibits CRC cell viability in a dose‐ and time‐dependent manner. Furthermore, this inhibitory effect is associated with the induction of ferroptosis, as BSN suppresses the cell viability of CRC by inducing GPX4‐regulated ferroptosis. BSN may bind to NRF2 protein to inactivate the NRF2 signaling, inhibiting the expression of p62 and HO‐1. Importantly, a low dose or a high dose of BSN significantly reduced the tumor growth in vivo.

**Conclusions:**

Our findings reveal that BSN blocks CRC growth by inducing p62/NRF2/GPX4‐regulated ferroptosis, which may be a novel lead compound for tumor treatment.

## Graphical abstract

BSN suppresses the cell viability of colorectal cancer by inducing GPX4‐regulated ferroptosis. BSN may bind to NRF2 protein to inactivate the NRF2 signaling, inhibiting the expression of p62 and HO‐1.

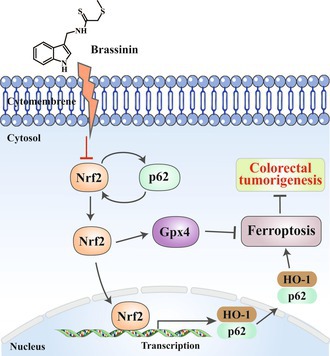

## 1 INTRODUCTION

Colorectal cancer (CRC) is the third most common cancer and ranks as the second deadliest cancer worldwide,^1^ and it is the most common cancer in the intestine.^2^ In recent years, the 5‐year survival rate of CRC patients has improved, but the death rate is still high.^3^ Chemotherapy, radiotherapy, or chemoradiotherapy are the preferred methods for the clinical therapy of CRC.^4^ However, drug resistance and adverse reactions pose significant challenges to the quality of life for CRC patients. Consequently, there is a pressing need to discover novel and effective drugs against CRC. Medications derived from plant resources that are also food homologues offer the advantages of high safety and the feasibility of long‐term use, which could potentially help to avoid tumor drug resistance.

Ferroptosis represents a mechanism of cancer cell death that can be induced by chemotherapeutic drugs. This process leads to toxic lipid peroxidation in cancer cells, ultimately resulting in cell death.^5^ Ferroptosis is primarily driven by the inhibition of the cystine/glutamate transporter (SLC7A11/xCT) and glutathione peroxidase 4 (GPX4). Notably, the level of Gpx4 in tumor from patients with advanced CRC has been found to be obviously higher than that in para‐cancer tissues.^6^ CRC cells exhibit insensitivity to ferroptosis, which may be associated with the frequent deficiency of Kelch‐like ECH‐associated protein 1 (KEAP1), a condition often observed in these cells.^7^ KEAP1 serves as the primary negative regulator of nuclear factor erythroid 2‐related factor 2 (NRF2).^8^ The deletion or mutation of KEAP1 disrupts the interaction between KEAP1 and NRF2, leading to NRF2 overexpression.^9^ NRF2, a key mediator of cellular oxidation reactions, has been confirmed to be intimately linked to ferroptosis resistance in cancer cells.^10^ In light of these findings, the activation of NRF2/GPX4‐regulated ferroptosis could suggest a resultful treatment strategy for CRC.

The rising incidence of CRC may be attributed to an increase in risk factors such as an aging population, alcohol and tobacco consumption, sedentary behavior, obesity, and a low intake of dietary fiber from sources like fruits and vegetables.^11^, ^12^ *Brassica campestris* L. var. campestris, belonging to the cruciferous family, is a commonly eaten vegetable in Asian countries. Indole phytoalexins, which are herbal compounds found in cruciferous vegetables, are known for their diverse anticancer effects.^13^, ^14^, ^15^ Brassinin (BSN), derived from *B. campestris* L. var. campestris, is believed to be a precursor to other phytoalexins.^16^ BSN has been shown to inhibit various types of cancer through a range of anticancer actions. For instance, BSN can block the metastasis in lung cancer,^17^ trigger cell apoptosis of prostate cancer,^18^ and suppress the proliferation of liver cells.^19^ However, whether ferroptosis mediates the anti‐CRC effect of BSN has not been previously studied. The paper sheds light on the role of BSN in inducing cell ferroptosis in CRC. The hypothesis is that BSN may induce cell ferroptosis in CRC by modulating the p62/NRF2/HO‐1 signaling pathway.

## 2 MATERIALS AND METHODS

### 2.1 Chemicals

BSN was obtained from Chengdu Push Bio‐Technology Co., Ltd., with a purity exceeding 98%. The compound was dissolved in DMSO to create a 1‐M stock solution to store for future use. When it is used, ensure that the final concentration of DMSO is less than 0.1% to minimize any potential effects on the cells. Deferoxamine (DFO), a ferroptosis inhibitor, was sourced from MedChemExpress (MCE) (product code HY‐D0903) and was dissolved in dimethylsulfoxide (DMSO) to create a 100‐μM stock solution.

### 2.2 Cell lines and cell culture

Human CRC cell lines RKO and HCT116 and the normal human colon cell line NCM460 were obtained from Cell Bank of the Chinese Academy of Sciences (Shanghai, China). They were cultured in Dulbecco's modified eagle medium (DMEM; Life Technologies, Gaithersburg, MD, USA) with 10% fetal bovine serum (FBS; Biological Industries, Israel) and 1% penicillin/streptomycin (GBCBIO Technologies, Guangzhou, China) at 37°C under 5% CO_2_.

### 2.3 Cell viability

The cell counting kit‐8 (CCK‐8; Dojido, Kumamoto, Japan) assay was used to determine the cell viability. Briefly, 2000–5000 cells were plated on a 96‐well plate and were exposed by BSN. After cell culture, 10‐μL CCK‐8 was added and incubated for 2 h. Then absorbance was detected at 450 nm using a microplate reader (PerkinElmer, USA).

### 2.4 Colony formation

According to previous research, we performed the colony formation assay.^20^ A total of 1000 cells were inoculated into a six‐well plate and incubated at 37°C for 14 days. The cells were then immobilized with 100% methanol, stained with 0.5% crystal violet, and colonies were counted by ImageJ software.

### 2.5 Identification of *B. campestris* L. ingredients

To collect the chemical ingredients of *B. campestris* L., we utilized the Traditional Chinese Medicine Systems Pharmacology Database and Analysis Platform (TCMSP, http://tcmspw.com/tcmsp.php) with the following filters: oral bioavailability (OB) ≥30% and drug‐likeness (DL) ≥0.18, and the Traditional Chinese Medicine Integrated Database (TCMID, http://119.3.41.228:8000/tcmid/search/) was applied. Furthermore, the PubChem (http://119.3.41.228:8000/tcmid/search/) was used for obtaining the canonical simplified molecular input line entry specification (SMILES) information of these ingredients.

### 2.6 Screening ingredient targets for *B. campestris* L.

The targets associated with bioactive ingredient of *B. campestris* L. were obtained from Swiss Target Prediction (http://www.swisstargetprediction.ch/). Swiss Target Prediction is a network server predicting potential targets by querying ingredients through reverse pharmacophore matching of internal pharmacophore model database. Only target with a norm probability >0.60 would be selected to guarantee the reliability of prediction (in Swiss Target Prediction).

### 2.7 Disease‐associated targets

Upload “colorectal cancer” as search terms to the DisGeNET (http://www.disgenet.org/search) and Genecards (https://www.genecards.org/, score ≥20). They reveal the relationship between targets and diseases from different perspectives.

### 2.8 Enrichment analysis

DAVID (https://david.nicifcrf.gov/) was used for gene ontology (GO) enrichment analysis with a *p* < 0.05 and *Kyoto Encyclopedia of Genes and Genomes* (KEGG) pathway enrichment analysis with a *p* < 0.05 and *q* < 0.05.

### 2.9 Molecular docking

The protein structure information was sourced from the Protein Data Bank (PDB, https://www.rcsb.org/). The compound structure was downloaded from the PubChem Compound database (https://pubchem.ncbi.nlm.nih.gov/). The criteria for protein selection were as follows: (1) the organism of origin is *Homo sapiens*; (2) the protein structure is determined using x‐ray crystallography; (3) the protein crystal resolution is 2.8 Å or better; (4) preference was given to protein structures that have been reported in molecular docking literature. Subsequently, molecular docking calculations were conducted using SYBYL‐X 2.0 software.

### 2.10 Lipid reactive oxygen species assay

The level of lipid reactive oxygen species (ROS) was measured using a C11‐BODIPY 581/591 kit (GC40165, Glpbio, USA). First, CRC cell lines were seeded in six‐well plates at a density of 6 × 10^5^/well. The cells were incubated with or without 200 μM of BSN treatment for 24 h. Then, they were incubated with C11‐BODIPY probe at a final concentration of 2 μM in a cell incubator for 30 min and then washed thrice by PBS. Subsequently, the cells were observed using a fluorescence microscope.

### 2.11 Western blot

The Western blot assay was carried out based on the previous research.^21^ First, Radio Immunoprecipitation Assay Lysis Buffer (RIPA) was used to extract the protein, and Bicinchoninic Acid Assay (BCA) was used to determine the protein concentration. Then total protein was separated using sodium dodecyl sulfate‐polyacrylamide gel electrophoresis (SDS‐PAGE) and adsorbed on polyvinylidene fluoride (PVDF) membranes by gel electrophoresis. To block the membranes, the membranes were immersed in 5% skim milk powder diluted by Tris‐buffered saline with Tween 20 (TBST). Primary antibodies were incubated at 4°C overnight, and the secondary antibody was incubated at room temperature for 2 h. Finally, the protein bands were exposed. Primary antibodies against GPX4 (1:1000), p62 (1:2000), NRF2 (1:1000), HO‐1 (1:1000), and the horseradish peroxidase (HRP)‐conjugated goat anti‐rabbit/mouse secondary antibody were obtained from Proteintech (Wuhan, Hubei, China). All antibodies were diluted with TBST.

### 2.12 Reverse transcription–quantitative polymerase chain reaction

The reverse transcription–quantitative polymerase chain reaction (RT‐qPCR) assay was performed following our previous experimental method.^21^ Total RNA was extracted from cells by the TRIzol reagent (TaKaRa, Osaka, Japan). RT‐qPCR was performed using a PrimeScript RT Reagent Kit (TaKaRa) and a SYBR Premix Ex Taq (TaKaRa) based on their instruction book. Gene expression data were analyzed using the 2^−∆∆Ct^ method.

The primer sequence is as follows:

p62/SQSTM1 (forward 5′‐CCGTCTACAGGTGAACTCCAGTCC‐3′, reverse 5′‐AGCCAGCCGCCTTCATCAGAG‐3′).

HO‐1 (forward 5’‐TCCTGGCTCAGCCTCAAATG‐3′, reverse 5’‐CGTTAAACACCTCCCTCCCC‐3′).

GAPDH (forward 5’‐CTCTCTGCTCCTCCTGTTCG‐3′, reverse 5’‐TGGCAACAATATCCACTTTACC‐3′).

### 2.13 Mouse xenograft model

Male 6‐week‐old BALB/c nude mice were procured from HUAFUKANG Bioscience (Beijing, China) and housed in the Laboratory Animal Research Center of Shanxi University of Chinese Medicine. Mouse experiments were approved by the Animal Ethical and Welfare Committee of Shanxi University of Chinese Medicine (permission number: AWE202403118). Tumor xenografts were established on the flanks of the mice through the subcutaneous injection of approximately 1 × 10^6^ RKO cells. One week postinjection, they were randomly assigned into three groups: a control group and two BSN‐treated groups receiving either a low dose (75 mg/kg) or a high dose (150 mg/kg) of BSN (*n* = 6 per group). The mice were intraperitoneally injected with BSN at doses of 75‐ or 150 mg/kg daily for 11 days. Tumor size was monitored daily using calipers, and tumor volume was calculated according to the formula: V = (length × width^2^)/2. After 11 days, the mice were euthanized, and the tumors were excised and weighed. Additionally, the heart, liver, spleen, lungs, and kidneys were individually weighed. Liver tissue samples was stained by hematoxylin and eosin (H&E).

### 2.14 Statistical analysis

All cell‐based assays were independently repeated thrice. Data are represented by mean ± standard error of the mean (SEM) of three independent experiments. Individual differences between the groups were analyzed by *t*‐test (two groups) or one‐way analysis of variance (ANOVA). Graphs were generated using Prism 9.0 (Graph Pad Software Inc. La Jolla, USA). The following were considered statistical significance between two groups: * *p* < 0.05, ** *p* < 0.01, ***p < 0.001, ****p < 0.0001.

## 3 RESULTS

### 3.1 BSN is the most important ingredient of *B. campestris* L. against CRC

**FIGURE 1:**
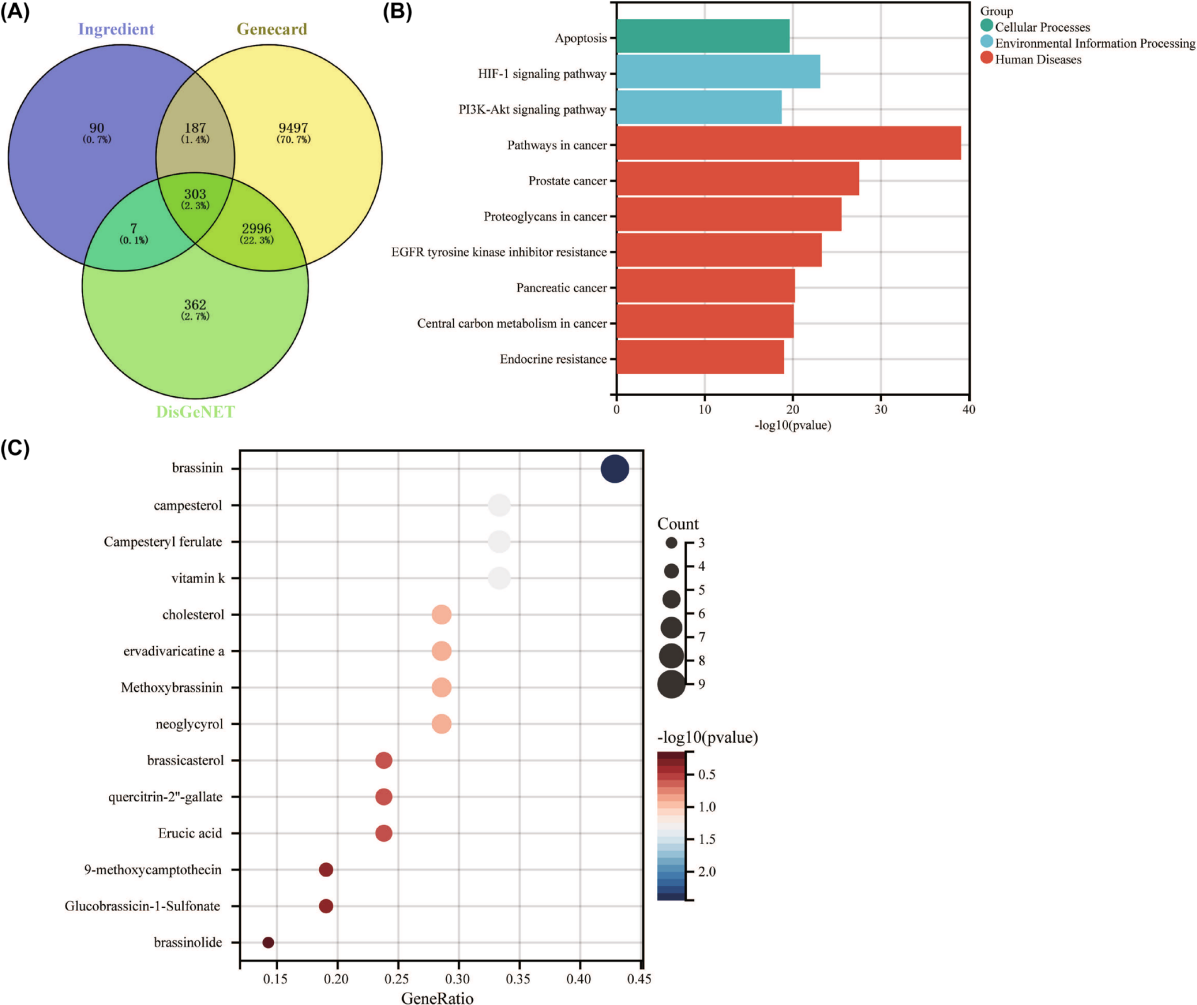
Brassinin (BSN) is the most important component of *Brassica campestris* L. against colorectal cancer. (A) Venn analysis about colorectal cancer–associated targets from Genecard and DisGeNET database and ingredient‐related targets in Brassica campestris L. (B) *Kyoto Encyclopedia of Genes and Genomes (*KEGG) enrichment analysis of 303 genes. (C) The bubble chart of the top 10 ingredients of brassicanine against colorectal cancer.

CRC‐associated targets from Genecard and DisGeNET database and ingredient‐related targets in *B. campestris* L. were analyzed by Venn. It was found that 303 genes are shared among them, and these genes are predictive targets for *B. campestris* L. treatment of CRC (Figure 1A). Further KEGG enrichment analysis of 303 genes revealed that pathway in cancer is the key pathway of *B. campestris* L. against CRC (Figure 1B), indicating that the components of *B. campestris* L. have certain antitumor effects. Finally, the important anti‐CRC ingredient in *B. campestris* L. was analyzed by a bubble chart. The result illustrated that BSN is the most important ingredient of *B. campestris* L. against CRC (Figure 1C). Therefore, we will proceed to study the anti‐CRC effect of BSN.

### 3.2 BSN demonstrates significant anticancer activity against CRC cells

**FIGURE 2:**
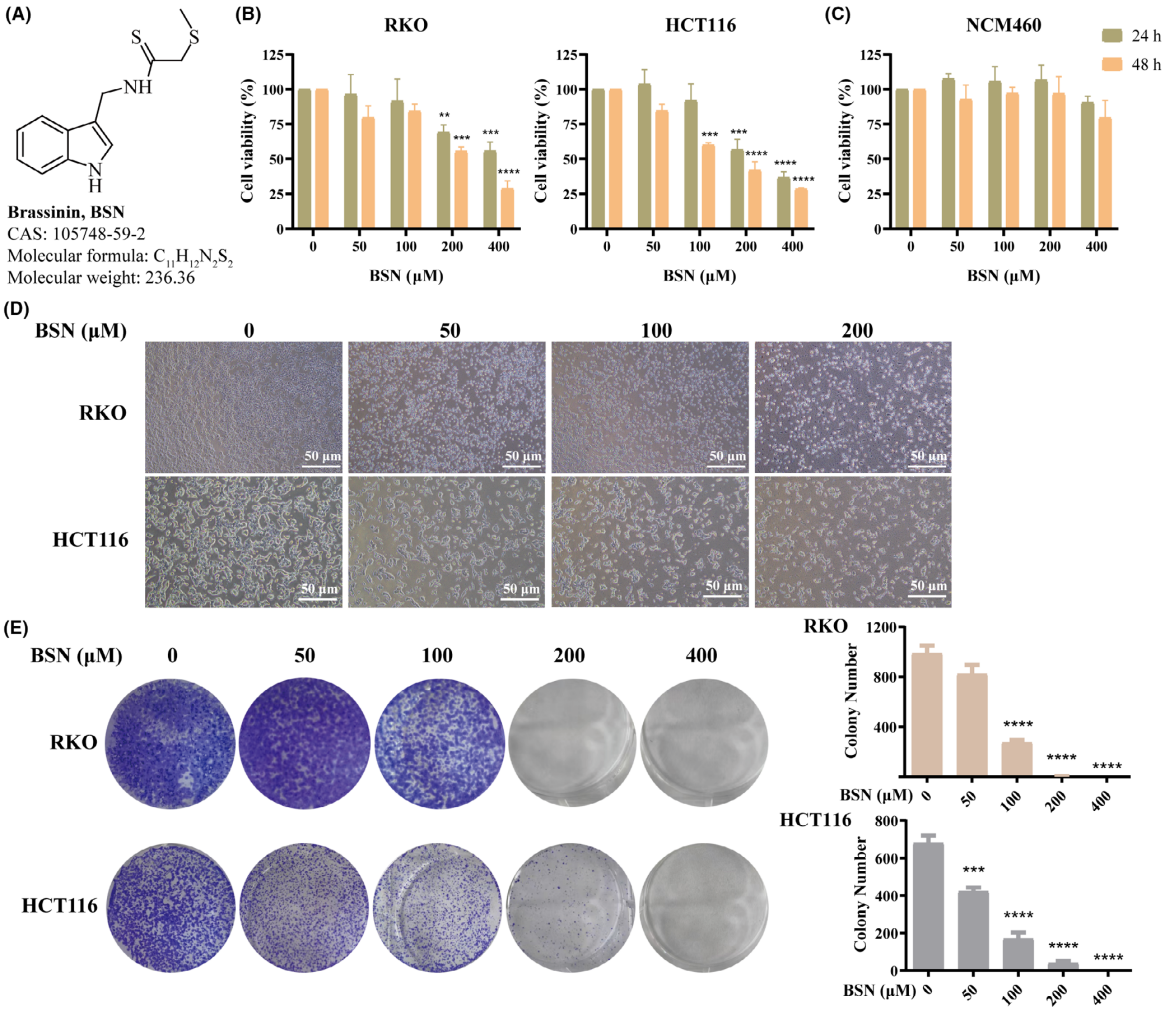
Brassinin (BSN) effectively suppresses the cell viability of CRC. (A) Chemical structure of BSN. (B) BSN suppressed the cell viability of CRC (RKO cell and HCT116 cell). (C) BSN was not cytotoxic to normal intestinal cells (NCM460 cell). (D) The morphological changes of RKO and HCT116 cells were observed by treatment with different concentrations of BSN under 10× magnification. (E) The effect of BSN on the cell colony formation of RKO and HCT116.. **p < 0.01, ***p < 0.001, ****p < 0.0001.

As depicted in Figure 2A, the molecular structure of BSN is presented. CRC cell viability, specifically RKO and HCT116, was assessed using the CCK‐8 assay after exposure to varying concentrations of BSN (1, 50, 100, 200, and 400 μM) for either 24 or 48 h. The findings indicated that the viability of both RKO and HCT116 cells was markedly reduced in a manner that was dependent on both the concentration and duration of BSN exposure (Figure 2B). Notably, a concentration of 100 μM BSN was observed to significantly diminish the viability of HCT116 cells after 48 h, whereas it did not substantially affect RKO cells. This suggests that HCT116 cells may exhibit greater sensitivity to BSN compared to RKO cells (Figure 2B). Importantly, the tested concentrations of BSN did not impact the activity of normal human intestinal epithelial cells, NCM460 (Figure 2C). Figure 2D shows that BSN, at different concentrations, gradually decreased the cell number of CRC and altered their morphology. Additionally, the colony formation assay revealed that BSN impeded the cell proliferation of CRC (Figure 2E). Collectively, these results indicate that BSN specifically inhibits the viability of CRC cells without exerting toxicity on normal cells. The dose 200 μM is IC50 of BSN that inhibits cell viability of RKO and HCT116, so the following study adopted 200‐μM dose of BSN for the antitumor study.

### 3.3 BSN induces ferroptosis in CRC cells

**FIGURE 3:**
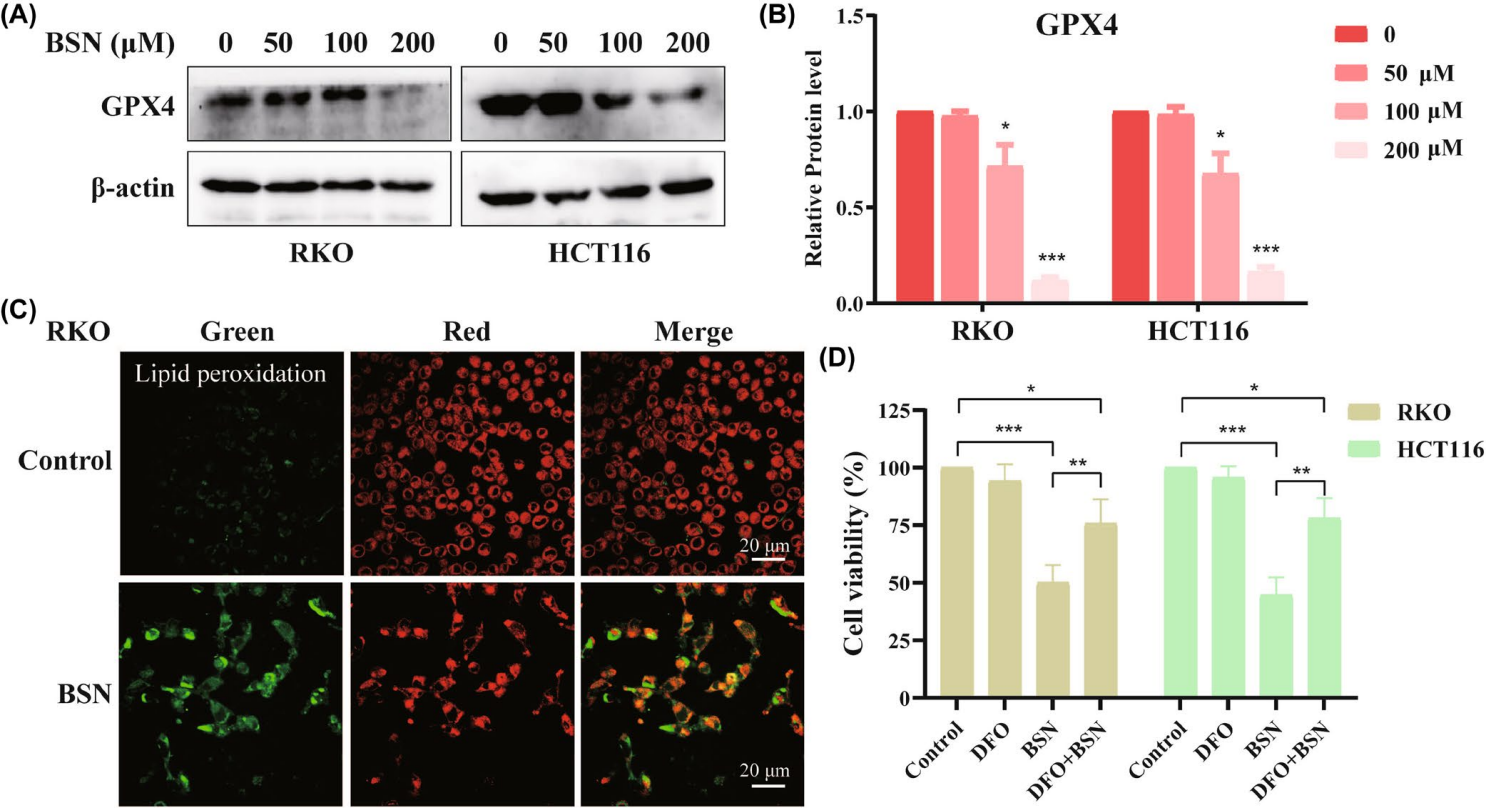
Brassinin (BSN) induces ferroptosis. (A) Western blot was performed to observe the protein level of GPX4 in RKO and HCT116 cells treated by different concentrations of BSN. (B) Statistical analysis of GPX4 protein level in (A). (C) The effect of BSN (200 μM) on inducing lipid peroxidation in CRC cells. (D) Colorectal cancer CRC cells were exposed to BSN (200 μM) in the absence or presence of deferoxamine (DFO, 100 μM), and then the cell viability was assayed. *p < 0.05, **p < 0.01, ***p < 0.001.

Ferroptosis, as a regulated form of cell death, is characterized by decreased GPX4 expression and is initiated by the inhibition of GPX4. Here, BSN was found to reduce the protein level of the GPX4 in both RKO and HCT116 cells in a dose‐dependent manner (Figure 3A,B). Importantly, in cells, lipid peroxidation occurs prior to the onset of ferroptosis, so lipid peroxidation was detected after BSN on CRC cells. The results showed that BSN significantly induced lipid peroxidation in CRC cells (Figure 3C). To further confirm whether BSN does induce ferroptosis, the effect of DFO, a ferroptosis inhibitor, was examined. The results indicated that DFO was able to restore cell viability in BSN‐treated CRC cell lines (Figure 3D). In conclusion, BSN can induce ferroptosis by inhibiting GPX4, thereby suppressing the viability of CRC cells.

### 3.4 BSN inhibits the p62/NRF2/GPX4 signaling pathway in CRC cells

**FIGURE 4:**
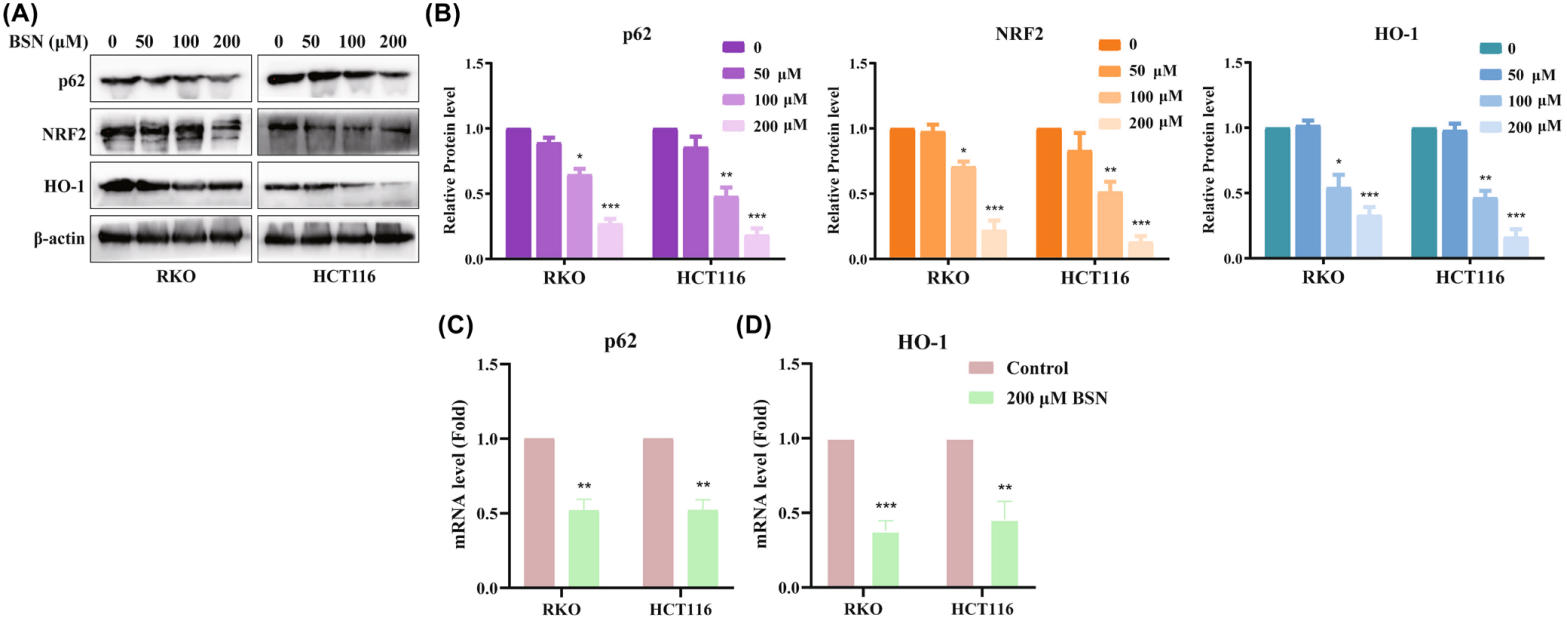
Brassinin (BSN) inhibits p62/NRF2/GPX4/HO‐1 signaling pathway. (A) Western blot was used to detect the expression level of p62, NRF2, and HO‐1 proteins in colorectal cancer (CRC) cells exposed by different concentrations of BSN. (B) Statistical analysis of expression level of p62, NRF2, and HO‐1 proteins in (A). (C, D) After treatment with 200‐μM BSN, reverse transcription–quantitative polymerase chain reaction (RT‐qPCR) assay tested the messenger RNA (mRNA) levels of p62 (C) and HO‐1 (D), which are Nrf2‐target genes. *p < 0.05, **p < 0.01, ***p < 0.001.

This research also explored the effect of BSN on the p62/NRF2/GPX4 signaling pathway in CRC cells. The protein levels of p62, NRF2, and HO‐1 were found to be downregulated in CRC cell lines treated with BSN (Figure 4A,B). Because p62 and HO‐1 are known target genes of Nrf2, the study further examined whether BSN could influence the messenger RNA (mRNA) levels of Nrf2 and HO‐1. RT‐qPCR assays confirmed that BSN significantly decreased the mRNA expression of p62 and HO‐1 in CRC cell lines, as shown in Figure 4C,D, respectively. Thus, BSN inhibits the p62/NRF2/GPX4 signaling pathway in CRC cells.

### 3.5 BSN may bind to NRF2 and GPX4

**FIGURE 5:**
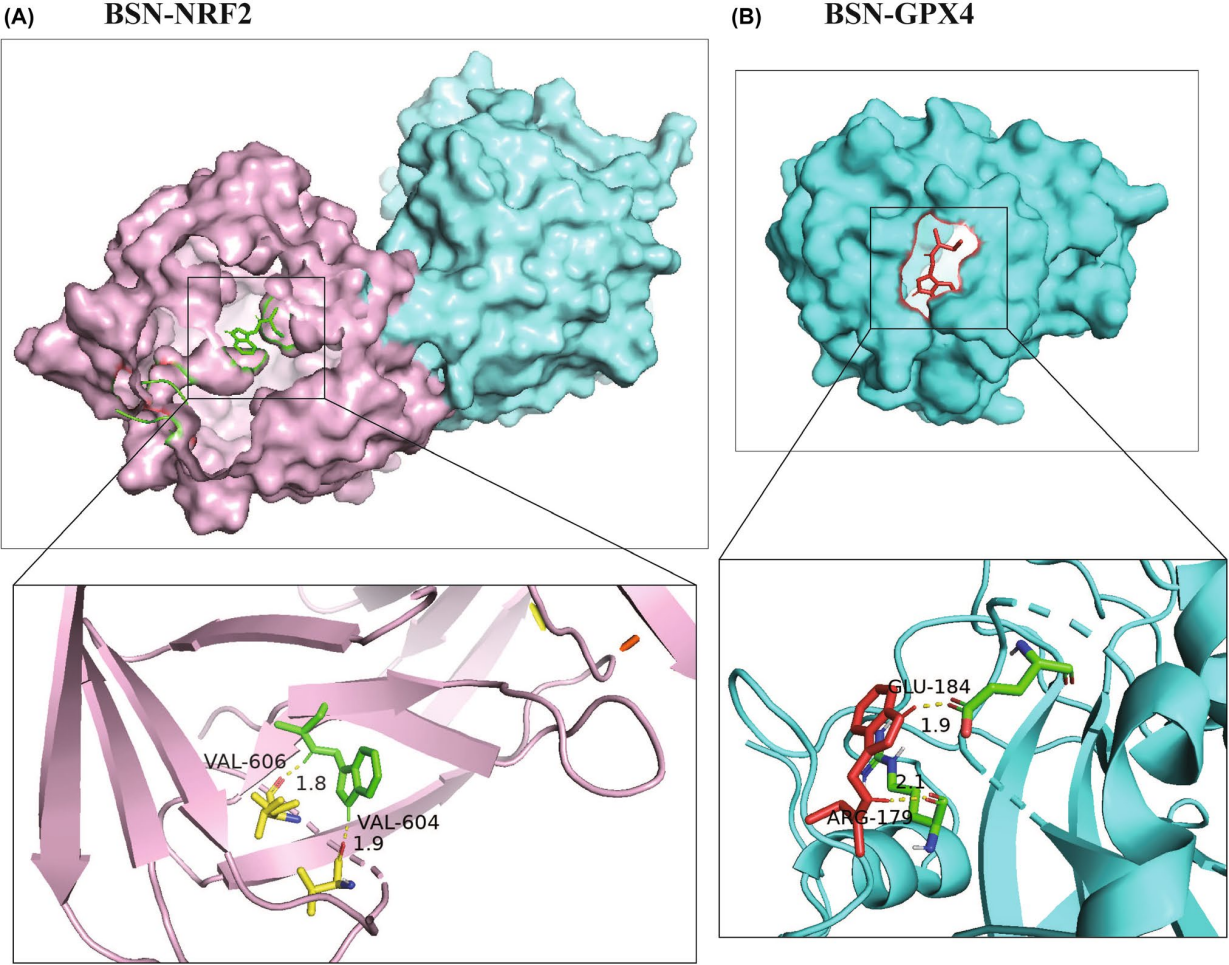
Brassinin (BSN) may bind to NRF2 and GPX4. (A, B) Molecular docking was performed to estimate the binding of BSN and NRF2 protein (A) or GPX4 protein (B).

Given that BSN can inhibit the protein levels of GPX4 and NRF2, resulting in the induction of ferroptosis, the study hypothesized that BSN might directly bind to these two proteins. To test this hypothesis, the binding affinity of BSN to NRF2 and GPX4 was assessed using molecular docking. The results indicated that BSN has a binding energy of −6.12 kcal/mol with NRF2 (Figure 5A) and a binding energy of −5.62 kcal/mol with GPX4 (Figure 5B). These scores suggest a potential binding interaction between BSN and the proteins NRF2 and GPX4, providing further insight into the mechanism by which BSN triggers ferroptosis in CRC cells.

### 3.6 BSN suppresses the growth of mouse xenograft

**FIGURE 6:**
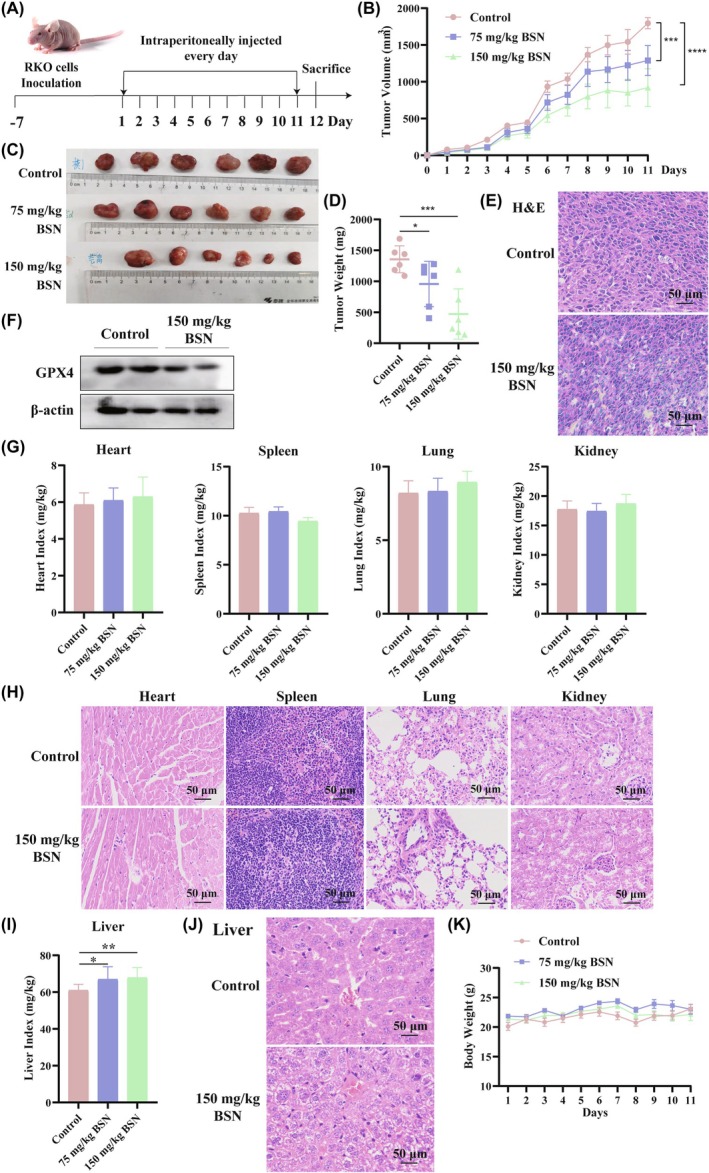
Brassinin (BSN) inhibits the growth of the mouse xenograft model. The RKO cell‐subcutaneous xenograft tumor mouse models were treated with intraperitoneal injection at 75‐ or 150 mg/kg BSN for 11 days (*n* = 6). (A) Plan of animal experiment. (B) Tumor growth curves were detected every day. (C) The tumor image from mice. (D) The tumor weight was measured. (E) Representative images of tumor hematoxylin and eosin (H&E) staining from mice that were injected intraperitoneally with control or 150‐mg/kg BSN. Scale bar: 50 μm. (F) The level of GPX4 protein was tested in tumor tissues using the Western blot assay. (G) The vital organ index (mg/g) was evaluated by measuring organ weight compared to body weight, including the heart, spleen, lung, and kidney. (H) Representative images of H&E staining from the heart, spleen, lung, and kidney of mice treated with control or 150‐mg/kg BSN. Scale bar: 50 μm. (I) Liver index. (J) Representative images of H&E staining from mice liver treated with control or 150‐mg/kg BSN. Scale bar: 50 μm. (K) Curve of body weight in xenograft tumor mice. *p < 0.05, **p < 0.01, ***p < 0.001, ****p < 0.0001.

Figure 6A shows the plan of animal experiment. The in vivo assay revealed that both the low and high doses of BSN slowed the growth of RKO‐xenograft tumors compared to the control group (*n* = 6). The tumors exhibited an obvious reduction in growth volume, size, and weight in the BSN‐treated groups, as depicted in Figure 6B–D, respectively. Concurrently, H&E staining evidenced that BSN significantly enhanced histological tumor damage (Figure 6E). Moreover, the level of GPX4 protein can be reduced by 150 mg/kg BSN in tumor tissues (Figure 6F). Thus, BSN has the potential to inhibit the growth of CRC in vivo by inducing ferroptosis.

To assess whether BSN has side effects, organ indices were measured. The indices for the heart, spleen, lungs, and kidneys showed no obvious variation among the two BSN‐treated groups and the control group (Figure 6G). Also, there was no difference in H&E staining of the heart, spleen, lung, and kidney tissues between the control group and the group treated with 150‐mg/kg BSN (Figure 6H). However, the liver index was found to be significantly increased following BSN treatment (Figure 6I). Subsequent H&E staining of liver tissues revealed pathological changes in the liver treated with 150‐mg/kg BSN compared to the control group (Figure 6J). In addition, during the course of the mouse xenograft treatment with BSN, the mouse body weight was detected daily. There was no significant variation in body weight across the three groups, as shown in Figure 6K. These results suggest that although BSN may have therapeutic potential against CRC, its effects on the liver require further investigation to ensure the safety of its application.

## 4 DISCUSSION

**FIGURE 7:**
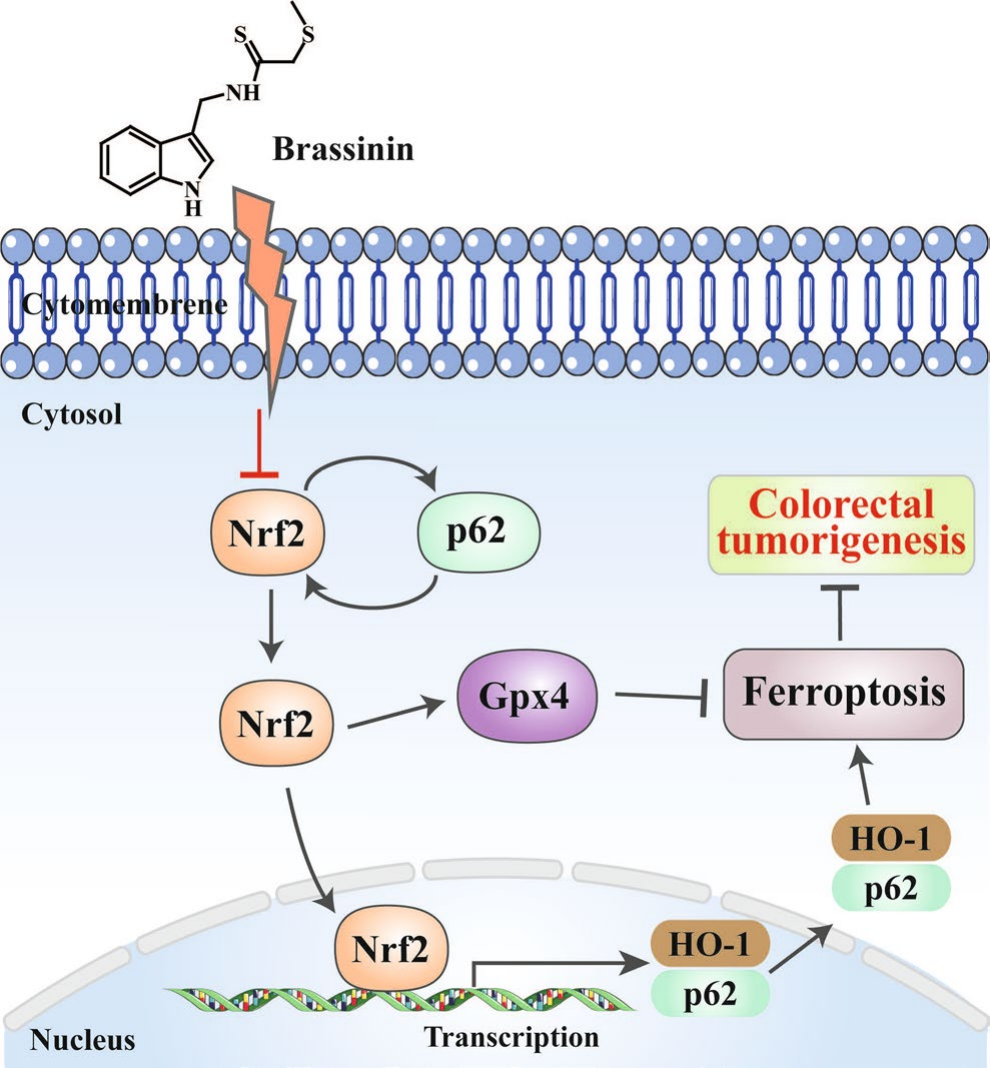
Brassinin (BSN) suppresses colorectal cancer (CRC) by inducing p62/NRF2/GPX4‐regulated ferroptosis. Ferroptosis is observed in BSN‐treated CRC. NRF2, a key transcription factor that negatively regulates ferroptosis, promotes GPX4 production and the transcription of p62 and HO‐1. BSN, binding to NRF2 protein, blocks the activity of NRF2 to induce cell ferroptosis, leading to the inhibition of CRC.

BSN is a potent phytoalexin naturally found in cruciferous vegetables and is known for its wide range of antitumor effects against various types of cancer. Our research has identified that BSN suppresses the growth of CRC through ferroptosis mediated by the inactivation of the p62/NRF2/GPX4 pathway (Figure 7). However, it has been discovered that BSN exhibits certain levels of hepatotoxicity. Although the antitumor activity of BSN has been documented in many reports, only one study has validated its antitumor effect in vivo without conducting a toxicity evaluation.^22^ Consequently, our study is the first to report on the hepatotoxicity associated with the use of BSN. Given its significant antitumor activity across various cancers, it is essential that future research focuses on further structural modifications or material encapsulation to mitigate the hepatotoxicity of BSN. This approach will aim to preserve the therapeutic benefits of BSN while reducing its potential adverse effects on the liver. By optimizing the delivery and bioavailability of BSN, it may be possible to enhance its safety profile, making it a more viable candidate for clinical application in cancer treatment. The development of strategies to minimize hepatotoxicity while maintaining the anticancer efficacy of BSN will be crucial for its translation from bench to bedside.

BSN enhances the expression of p21 and p27, thereby inducing G1 phase arrest in the cell cycle of human colon cancer cells.^23^ Additionally, the BSN derivative homobrassinin exerts an inhibitory effect on the proliferation of human CRC Caco2 cells using a ROS‐dependent antiproliferative mechanism.^24^ However, the mode of cell death induced by BSN has not been discovered and reported in CRC. In some other cancers,^18^, ^25^ BSN has been reported to promote apoptosis, but it has not been found to induce ferroptosis in tumor cells. Ferroptosis is a form of programmed cell death that is mediated by iron dependency and the deposition of lipid ROS. Numerous studies have indicated that the evocation of ferroptosis can effectively block cell proliferation in many tumors.^26^ This process is characterized by the inhibition of GPX4 and the deposition of intracellular lipid ROS.^27^ Several reports have shown that GPX4 is a downstream target gene of NRF2, and the suppression of NRF2 represents a promising way for antitumor chemotherapy.^10^ NRF2 is a key regulator of the antioxidant response and promotes its anti‐ferroptotic effect by modulating the expression of its target genes like HO‐1and SLC7A11.^28^ Among these, HO‐1 is a key gene that Nrf2 targets, commonly used to gauge the activation of the NRF2 pathway. Many reports have proved that inhibiting NRF2 signaling can enhance the sensitivity of tumor cell to ferroptosis, whereas activating NRF2 signaling can offer protection against this form of cell death.^6^ The activated NRF2 signaling pathway may initially guard cancer cells during the early stages of tumor development but can later block tumor metastasis and progression.^29^ In advanced CRC, the upregulation of NRF2 is linked to an increase in antioxidant proteins like GPX4, which could mitigate lipid peroxidation and thereby inhibit ferroptosis.^7^ Therefore, targeting NRF2 and GPX4 could be an efficient strategy to induce ferroptosis in CRC. Our research revealed that BSN can bind to NRF2 and GPX4 proteins and suppress their expression. This action by BSN promotes ferroptosis in CRC cells, presenting a potentially powerful strategy to effectively treat this disease. By targeting these key proteins, BSN may offer a novel avenue for enhancing the efficacy of anticancer treatments by leveraging the mechanisms of ferroptosis.

Protein p62, a scaffold protein, is involved in processes such as tumorigenesis, signal transduction, and responses to oxidative stress. It plays a major role in the NRF2 signaling by facilitating the depolymerization of the NRF2‐KEAP1 complex.^27^ During the oxidative stress associated with ferroptosis, cancer cells activate p62, which in turn stimulates the autophagic degradation of KEAP1. This leads to a positive feedback loop that maintains the continuously activated NRF2 signaling, aiming to preserve oxidative homeostasis.^27^, ^30^ The activated NRF2 is instrumental in the upregulation of various genes that prevent ferroptosis, including GPX4.^31^ Research has indicated that p62 acts as an oncogene in CRC.^32^ It is interesting that elevated p62 expression is necessary to activate NRF2,^33^ and that p62 is a target gene of Nrf2, suggesting the existence of a positive feedback loop within the p62‐NRF2 axis.^32^ Our study showed that BSN can suppress the expression level of p62, leading to the inactivation of NRF2. This suppression subsequently reduces the mRNA levels of p62, ultimately inhibiting the growth of CRC. Consequently, BSN exerts its anticancer effects through inhibition of the p62‐NRF2‐HO‐1 pathway, thereby inducing ferroptosis in CRC cells. This finding provides greater insight into the molecular mechanisms through which BSN may be utilized as a potential therapeutic agent against CRC.

This study, while providing valuable insights, has several limitations that warrant acknowledgment. First, the investigation utilized only one ferroptosis inhibitor and did not employ any NRF2 agonist to fully evaluate the role of BSN in NRF2‐regulated ferroptosis. This approach limits the comprehensiveness of the findings regarding the mechanisms of action of BSN in this context. Additionally, the study did not assess whether BSN can be beneficial to the levels of lipid peroxidation, a hallmark of ferroptosis. Understanding the impact of BSN on this key process is crucial for elucidating its role in inducing ferroptosis and its capacity as a therapeutic agent. Lastly, this paper did not validate reasons by which BSN inactivates the p62/NRF2/HO‐1 pathway to induce ferroptosis in CRC within in vivo models. Addressing these limitations will be crucial for the advancement of BSN as an effective therapeutic strategy for CRC.

## 5 CONCLUSION

In conclusion, we demonstrate that BSN induces ferroptosis through the inactivation of the p62/NRF2/HO‐1 pathway. It is needed to further explore the mechanism by which BSN inhibits the p62/NRF2/HO‐1 pathway and to investigate the potential for BSN to induce ferroptosis in CRC through additional pathways or mechanisms. This deeper understanding will be crucial for the development of BSN as a potential therapeutic strategy of CRC.

## AUTHOR CONTRIBUTIONS

**Shi‐Yuan Wen:** Conceptualization; data curation; formal analysis; funding acquisition; methodology; project administration; resources; software; visualization; writing – original draft; writing – review and editing. **Rui‐Rui Gao:** Data curation; formal analysis; investigation; software; validation. **Yan‐Yan Chen:** Data curation; investigation; methodology; software; visualization. **Yi‐Jie Wang:** Investigation; methodology; software. **Xin‐Tong Wang:** Data curation; formal analysis. **Hai‐Xin Liu:** Conceptualization; funding acquisition; project administration; resources; writing – review and editing.

## CONFLICT OF INTEREST STATEMENT

The authors declare that there are no conflicts of interest.

## FUNDING INFORMATION

This work was supported by Natural Science Foundation of Shanxi Province for Youths (numbers: 20210302123310 and 20210302124668), Science Research Start‐up Fund for Doctor of Shanxi Medical University (number: XD2021), Science and technology innovation ability cultivation program project of Shanxi University of Chinese Medicine (number: 2022PY‐TH‐17), and the immune regulation Chinese medicine research and development innovation team project (number: 2022TD1017).

## ETHICS STATEMENT

All animal experiments were conducted in accordance with the principles of good laboratory animal care and performed in compliance with the Animal Ethics Review Committee of Shanxi University of Chinese Medicine.
